# Preschoolers’ attention to and learning from on-screen characters that vary by effort and efficiency: An eye-tracking study

**DOI:** 10.3389/fpsyg.2022.1011172

**Published:** 2022-12-15

**Authors:** Koeun Choi, Molly A. Schlesinger, John M. Franchak, Rebekah A. Richert

**Affiliations:** ^1^Department of Human Development and Family Science, Virginia Polytechnic Institute and State University, Blacksburg, VA, United States; ^2^Department of Psychology, University of California, Riverside, Riverside, CA, United States

**Keywords:** selective social learning, effort, efficiency, visual attention, eye-tracking, media characters

## Abstract

Prior findings are mixed regarding the extent to which children understand others’ effort in early childhood. Especially, little is known about how character effort impacts children’s selective attention and learning. This study examined preschoolers’ visual attention to and learning from two on-screen characters: One character exerting high effort with low efficiency and another character exerting low effort with high efficiency in solving problems successfully. Children between 3.5 and 6.5 years of age (*N* = 70) watched a video of the two on-screen characters successfully solving problems. Children’s eye movements were recorded during viewing. Each of the two on-screen characters consistently displayed either high effort/low efficiency or low effort/high efficiency to solve four problems (familiarization). For the final problem (testing), the two characters exerted the same level of effort as each other and used unique solutions to solve the problem. Children then solved the final problem themselves using real objects. Children could selectively use either character’s solution demonstrated in the video. Lastly, children explicitly judged how good the characters were at solving problems. Younger children were more likely to use the solution demonstrated by the character with high effort/low efficiency, whereas older children were more likely to use the solution provided by another character with low effort/high efficiency. Younger children allocated more attention to the high effort/low efficiency character than the low effort/high efficiency character, but this pattern was modified by age such that children’s gaze to the low effort/high efficiency character increased with age. Children’s explicit credibility judgments did not differ by character or child age. The findings are discussed with respect to preschoolers’ understanding of effort and implications for children’s learning from screen media.

## Introduction

Screens are ubiquitous in children’s lives (Rideout and Robb, 2020). The prevalence of screen media has been further intensified by rapid technological and social changes, such as the COVID-19 pandemic (Hartshorne et al., 2021). Thus, educationally-focused media provide an increasingly important context for young children’s learning. Given the social nature of children’s observations and imitations, on-screen informants may be particularly well-positioned to capitalize on the processes involved in observational learning (Richert et al., 2011). A growing body of literature hypothesizes relations between preschoolers’ perceptions of on-screen characters and learning (Calvert et al., 2007; Lauricella et al., 2011; Richert et al., 2011; Bond and Calvert, 2014). Indeed, emerging evidence suggests the role of media characters in children’s learning in early childhood (Schlesinger et al., 2016; Calvert et al., 2020). However, further empirical research is needed to understand how specific character traits, such as character effort, are perceived by preschoolers and whether these perceptions guide children’s attention to and learning from media characters. The current experimental study addresses this gap in the literature by examining the impact of character effort when it is inversely related to efficiency on preschoolers’ selectivity in their visual attention to and learning from on-screen characters.

### Preschoolers’ understanding of character effort

Beliefs about effort play a critical role in motivation and achievement (Dweck, 2006; Yeager and Dweck, 2012). Studies with school-aged children and adults have revealed that individuals are more like to persevere through challenging tasks and remain motivated if they believe that hard work is essential for success rather than reflecting a lack of abilities (Dweck and Leggett, 1988; Dweck et al., 1995; Blackwell et al., 2007). Children’s reasoning about effort in early childhood is likely to lay the groundwork for developing beliefs about the value of hard work in middle childhood years and beyond (Muenks et al., 2018; Leonard et al., 2021).

However, prior findings present a mixed picture of children’s understanding of effort in early childhood. Some studies suggest that young children believe in a positive relation between effort and ability, thinking that smart people work hard, persevere, and exert high effort (Nicholls, 1978, 1979; Nicholls and Miller, 1984; Stipek and Gralinski, 1996; Folmer et al., 2008). For example, Nicholls (1978) showed children between 5 and 13 years of age a series of films, depicting a pair of actors solving math problems side by side. In these films, one actor spent the entire time working on math problems. The other actor completed them early and spent the remaining time on task-irrelevant activities, but both actors received identical scores. After watching videos, the 5–9-year-old children rated the actor exerting high effort as smarter than the low-effort actor. In contrast, the 10–13-year-old children rated the low-effort actor as smarter than the high-effort actor. Nicholls (1978) interpreted the younger children’s reasoning as a consequence of an immature concept of effort, due to difficulties in understanding the compensatory relation between effort and ability. Based on these findings, some scholars have posited that younger children’s seemingly irrational but optimistic view about effort may lead young children to be more effort-oriented and resilient to failure (e.g., Nicholls and Miller, 1984; Dweck et al., 1995).

In contrast, evidence from other studies challenges the assumption that preschoolers’ reasoning is effort-oriented. When using tasks that are less cognitively taxing, researchers found that preschoolers aged 3–5 years made adult-like inferences about others’ abilities based on how hard these characters tried or how difficult the characters felt about given problems (Wimmer et al., 1982; Heyman et al., 2003). With concrete examples and explicit visual cues, 4-year-old children were able to infer high effort from a combination of low ability and high outcome (Wimmer et al., 1982). Moreover, when provided with information about each character’s perceived task difficulty, 3-year-old children considered a character who found a task to be easy as being smarter than another character who found the same task to be hard (Heyman et al., 2003). In line with these findings, Cimpian (2017) argued that preschoolers may not be as optimistic and resilient as previously believed. In other words, young children’s adult-like reasoning—that effort suggests a lack of skill—may place them at risk for adverse outcomes such as low self-esteem and helplessness, similar to older children and adults.

Indeed, recent research has revealed that young children make sophisticated decisions on when and how hard they try (Sommerville et al., 2018; Leonard et al., 2020, 2021; Lucca et al., 2020). Throughout a series of experiments, Leonard et al. (2020) have shown that preschoolers aged 4 and 5 years calibrate their level of effort by taking into account multiple aspects related to effort allocation. In these experiments, the effort of an adult model influenced children’s effort only when the adult succeeded but not when the adult failed. Moreover, children worked hardest when the adult succeeded after testifying about the value of effort and producing effortful actions. Similarly, studies with infants have found that children before their second birthday allocate effort after considering various factors, including others’ effort, the utility of effort, the difficulty of tasks, and the capability of children themselves (Sommerville et al., 2018; Lucca et al., 2020). Together, these studies have shown that young children not only carefully observe the level and utility of others’ effort but also systematically allocate their effort in consideration of task difficulty and the value of effort, contradicting the claim that young children’s reasoning about effort is irrational.

However, the findings on children’s rational decisions about effort allocation do not eliminate the possibility of young children’s optimism about effort (Wimmer et al., 1982; Leonard et al., 2020, 2021). In one of the experiments of Leonard et al. (2020), an adult model introduced a task as being difficult for children and then completed the task either effortfully or effortlessly. Despite the expected task difficulty, 4–5-year-old children exerted effort regardless of the adult’s effort as long as the adult had succeeded at the task. Furthermore, Leonard et al. (2020) found across all experiments that children spent some time trying to solve the given problem rather than giving up immediately, even after seeing the adult work hard and fail. These patterns are in contrast to the findings from adults and older children: Adults and 5 and 6th graders are less likely to choose or perform well when tasks are labeled as “hard” than “easy” (Hom and Maxwell, 1983; Scasserra, 2008). Thus, compared to adults and older children, young children appear to be more willing to put forth effort even when facing a challenge. Similarly, Wimmer et al. (1982) found that children aged 4, 6, and 8 years allocated higher reward to a character showing high effort and low ability compared to another character showing low effort and high ability regardless of outcome even though these children accurately inferred relations among effort, ability, and outcome. These findings suggest that early childhood could be a period where children tend to be hopeful and confident about the outcome of effort. Yet, it remains to be seen how this pattern changes over development.

### Preschoolers’ understanding of character effort given inversely related efficiency

Prior research has indicated that developmental changes in children’s reasoning about effort should be understood in the context of associated factors such as quality of outcome or task difficulty (Wimmer et al., 1982; Sommerville et al., 2018; Leonard et al., 2020; Lucca et al., 2020). Another factor that is closely linked to the relation between effort and outcome is efficiency, the ability to maximize outcomes at minimum costs. From infancy, children are sensitive to efficiency, preferring efficient solutions compared to inefficient ones (Gergely and Csibra, 2003; Jara-Ettinger et al., 2015; Liu and Spelke, 2017; Liu et al., 2019; Colomer et al., 2020; Colomer and Woodward, 2023). Infants as young as 3 months calculate relative costs associated with different actions and expect others to prefer low-cost actions to high-cost actions (Liu et al., 2019). Further, 18-month-old infants selectively pay attention to and learn from an efficient actor rather than an inefficient actor (Colomer and Woodward, 2023). Similarly, preschoolers interpret the costs required to perform different actions and systematically use that information to judge others (Jara-Ettinger et al., 2015).

Prior research on preschoolers’ perceptions of effort has mainly focused on the inverse relation between effort and efficiency (Wimmer et al., 1982; Leonard et al., 2019). For example, Wimmer et al. (1982) examined how children aged 4, 6, and 8 years allocated rewards based on others’ effort, ability, and performance. In doing so, researchers contrasted high effort/low ability versus low effort/high ability. This confound was intentionally introduced to avoid young children’s confusion about a mismatch between effort and ability (Wimmer et al., 1982). Without levels of ability that explained the discrepancy between effort and outcome, researchers found that children were confused about understanding a combination of low effort and high outcome or a combination of high effort and low outcome. Leonard et al. (2019) also found that 4-year-old children did not systematically distinguish levels of effort when the outcome was perceptually matched between high and low effort characters.

Thus, although it is possible for someone to work hard to solve problems efficiently or work effortlessly but remain inefficient, the current study covaried levels of efficiency with levels of effort. This method allows for the comparison between high and low effort characters in consideration of their contrasting levels of efficiency as a potential explanation of the discrepancy between different levels of effort and the same performance outcome. In terms of outcomes, the current study focuses on success rather than failure based on prior research showing the significant impact of adult model’s effort on preschoolers’ effort only with successful outcomes (Leonard et al., 2020). Thus, in this study, we focus on children’s understanding of high and low effort characters with inversely related efficiency levels when the characters’ performance outcomes are equally successful.

### Preschoolers’ selective social learning as a function of character effort

Children are often exposed to information from more than one source, such as parents, siblings, peers, and media characters; and children need to decide whom to pay attention to and learn from. Although emerging evidence exists to indicate young children’s rational thinking regarding effort allocation (Sommerville et al., 2018; Leonard et al., 2020; Lucca et al., 2020), each child in those studies observed effort at a constant level, which was either high or low but not both. When such comparison is allowed, children may weigh the amount of effort each character deploys and begin to form preferences. For example, Vanderbilt et al. (2014) found that 3–4-year-old children were willing to learn information from an inaccurate character only when there was no conflicting information provided from an accurate character. Although it remains a question whether young children would consider effort as an indicator of characters’ credibility to guide their learning, a large body of research has documented that preschoolers are sensitive to other characteristics that determine which type of individual makes trustworthy claims (Mascaro and Sperber, 2009; Shafto et al., 2012; Mills, 2013).

More than a decade of research on selective social learning has demonstrated that young children monitor the trustworthiness of informants and make deliberate choices when learning from others, referred to as *selective trust* (see Harris, 2012 for review). After being exposed to two informants providing contrasting information, preschoolers overwhelmingly choose to learn from accurate informants over informants who have previously made errors (Koenig and Harris, 2005). When children have little information about the past reliability of informants, children as young as 3 years of age selectively learn from an individual who expresses confidence rather than one who indicates uncertainty (Jaswal and Malone, 2007) and recognize informants have specific expertise and knowledge on particular subjects (Lutz and Keil, 2002). Children between 3 and 5 years are also sensitive to others’ intent, preferring to learn from individuals who are benevolent (Landrum et al., 2013), honest (Mascaro and Sperber, 2009), and helpful (Vanderbilt et al., 2014).

Prior research has examined children’s selective trust using both implicit and explicit measures of children’s learning preferences (Corriveau and Harris, 2009; Corriveau et al., 2016; Leech et al., 2019). Children’s implicit preferences have been measured by asking children to selectively endorse the information (e.g., labels and problem solutions) presented by one of the informants. On the other hand, children’s explicit preferences have been assessed through children’s direct judgments of the credibility of the informants (e.g., which informant was better or how good each information was at answering questions; Bascandziev and Harris, 2016; Leech et al., 2019). Given that implicit and explicit measures do not always yield symmetric results (Bascandziev and Harris, 2016; Corriveau et al., 2016; Leech et al., 2019), it is important to implement both measures to better understand the role of character effort in children’s learning from social partners. Exploring any potential asymmetry between these measures may contribute to explaining the differences in preschoolers’ understanding of high and low effort characters in the literature.

### Preschoolers’ visual attention as a function of character effort given inversely related efficiency

Preschoolers’ selective attention based on character effort may be observed earlier than when children are asked to make use of the information provided by characters. Koenig and Sabbagh (2013) have suggested that selectivity in social learning can be manifested in selective encoding or selective expression of information presented by informants. As researchers have noted the importance of examining cognitive processes driving children’s social learning (Koenig and Sabbagh, 2013; Sobel and Kushnir, 2013; Sabbagh et al., 2017; Mangardich and Sabbagh, 2018), eye-movement research has begun to capture the ongoing dynamics of selective social learning in real time, examining whether selectivity would exhibit in the ways in which children encode information from different characters or in the extent to which children recall and use what they have learned from the characters.

Prior eye-movement studies have revealed that children from infancy selectively allocate attention based on others’ reliability. Through two eye-tracking experiments, Tummeltshammer et al. (2014) showed that 8-month-old infants looked longer at the location that was gazed at by a statistically reliable human informant than the location that was gazed at by a statistically unreliable human informant. Similarly, Chow et al. (2008) found that 14-month-old infants were less likely to follow the gaze of a previously unreliable looker who smiled excitedly at an empty box than a previously reliable looker who showed the same reaction to a box containing a toy. These findings suggest that even young children can monitor character reliability and selectively decide what they should attend to.

Some eye-tracking studies with preschoolers that incorporated both eye-tracking and behavioral measures have shown consistency between children’s visual attention and behavioral performance in selective social learning tasks (Sobel et al., 2012; Howard et al., 2015). Sobel et al. (2012) found that older 3-year-old (41–47 months) and 4-year-old children’s gaze to novel objects was longer when the objects were introduced by an informant with a history of accurate labeling than when presented by an informant who inaccurately labeled the object (Sobel et al., 2012). Similarly, when the previously inaccurate informant presented a novel label, 3- and 4-year-old children avoided looking at the object the informant gazed at and instead looked at the other object. Thus, these studies have shown that preschoolers’ eye movements and inferences about the novel words parallel each other, both informed by the past accuracy of the characters.

However, emerging evidence suggests that the patterns of visual attention during social learning may vary with age (Howard et al., 2015; Barry-Anwar et al., 2017). Howard et al. (2015) measured preschoolers’ imitation of in-group and out-group on-screen characters and children’s eye gaze to these characters during viewing. Younger 3-year-old (36–41 months) children looked equally to both characters, but these children were more likely to imitate the in-group character than the out-group character. As children’s ability to disengage from salient distractors improves with development (Colombo and Cheatham, 2006), this finding suggests that the social cues from the characters, regardless of the conditions, may be too salient to ignore for this age group.

Children’s voluntary control of attention continues to develop through early childhood. Indeed, some eye-tracking studies of dynamic scene (video) viewing show age-related increases in looking to relevant areas on screens (Frank et al., 2009; Kirkorian et al., 2012; Franchak et al., 2016; Kirkorian and Anderson, 2018). Yet, others find no consistent age-related changes (Kadooka and Franchak, 2020; Kirkorian et al., 2022). Using a wide age range and a diverse set of videos, Kadooka and Franchak (2020) revealed that developmental differences in visual attention to socially relevant features were constantly changing with specific parts of the videos, rather than occurring solely as a function of age, suggesting a need to take into account specific contexts that help understand which information on a scene is meaningful for viewers of different ages. Together, prior studies highlight the importance of considering age-related differences when examining preschoolers’ visual attention in the particular context of selective social learning from high and low effort on-screen characters given inversely related efficiency.

### Overview of the current study

The purpose of the current study was to examine preschoolers’ attention to and learning from high vs. low effort characters with inversely related efficiency. Preschoolers (3.5–6.5 years) watched a video including a character who tried hard using inefficient solutions (high effort/low efficiency) and another character who easily solved the problem using efficient solutions (low effort/high efficiency). Children’s eye movements were recorded during video viewing. After watching the video, children were asked to solve the last problem using real objects and to rate each character’s credibility.

Given mixed findings in prior research, it remained an open research question whether character effort with inversely related efficiency impacts children’s visual attention as well as implicit and explicit learning preferences and the extent to which age moderated the effects of character effort. If preschoolers understand the compensatory relation between effort and ability in a similar manner as adults (Wimmer et al., 1982; Heyman et al., 2003; Cimpian, 2017), children would prefer those who work effortlessly using efficient solutions by paying greater attention, giving a higher credibility rating, and showing a higher likelihood of learning from the low effort/high efficiency character compared to the high effort/low efficiency character. Alternatively, preschoolers may show effort-based reasoning by preferring those who work hard and inefficiently (Nicholls and Miller, 1984; Folmer et al., 2008).

## Materials and methods

### Participants

Participants were 70 children aged 3–6 years (*M* = 5.10, *SD* = 0.82, *range* = 3.67–6.71, 36 boys and 34 girls). Child ethnicity (*n* = 68) was reported by 97% of the families, and participants were diverse in ethnicity: 34% Hispanic/Latino & White Caucasian, 25% While/Caucasian, 22% Hispanic/Latino, 4% Asian & White Caucasian, 3% Hispanic/Latino & Black/African American, 3% Hispanic/Latino & American Indian/Alaska Native/Native Hawaiian/Pacific Islander, 4% Multiple, 1% White Caucasian & American Indian/Alaska Native/Native Hawaiian/Pacific Islander, 1% Black/African American, and 1% Asian. Further, parents had varying levels of education and income. Of the 96% of parents (*n* = 67) who reported their highest degree completed, 4% did not complete high school, 11% completed high school or GED, 43% completed some college or an associate’s degree, 25% had a bachelor’s degree, and 17% had an advanced degree. Among the 91% of parents who reported their yearly income levels (*n* = 64), 23% had earnings less than $29,999, 19% of families had $30,000–49,999, 14% had $50,000–69,000, 17% had $70,000–89,000, 16% had $90,000–149,000, and 11% had more than $150,000.

Families were from Southern California, and they were recruited through a laboratory database, community flyers, and community events. Child participants and their caregivers visited the lab for one 60-min session. To participate, English had to be the primary language spoken at home. Children received a small toy, and parents received $20 compensation for travel costs. One additional participant was excluded from analyses because of failure to complete all tasks. The Institutional Review Board approved the experimental protocol, and informed consent was obtained from a caregiver of each child.

To determine the sample size of the study, we reviewed existing studies on preschoolers’ selective learning, including behavioral and eye-tracking measures (Koenig and Harris, 2005; Jaswal and Malone, 2007; Sobel et al., 2012). A medium to large effect size was observed from the results of the previous studies. A sample of 48–66 subjects would be required to detect an effect size of *f*^2^ = 0.15 to 0.20 with α = 0.05 and power = 0.8 when three variables are included in either a logistic regression model or a linear mixed-effects model, according to power analyses using *pwr.r.test* function from the pwr package (version 1.3-0; Champely, 2018).

### Video stimulus

**Figure 1:**
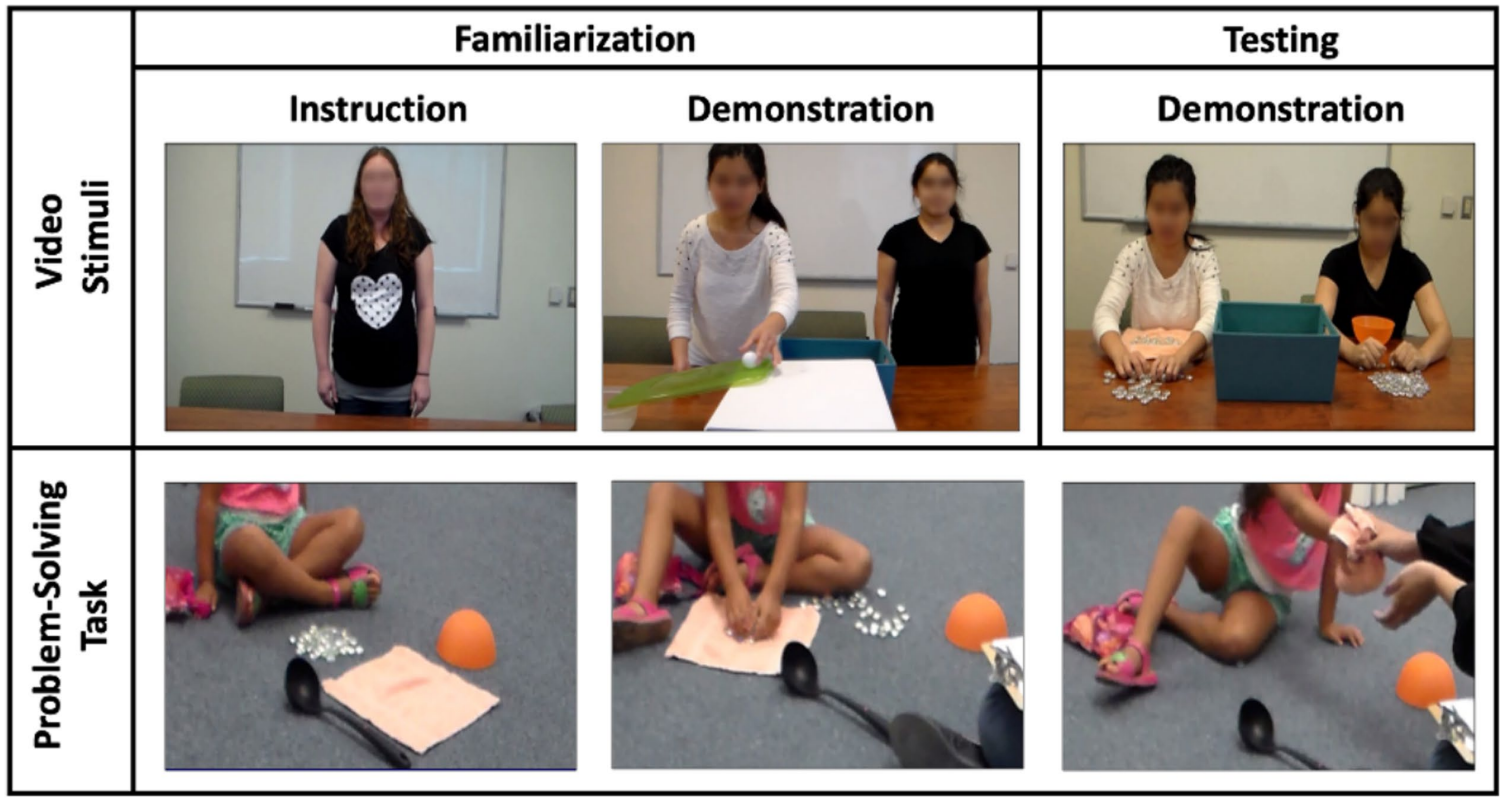
Stimulus video (top) and problem-solving task procedure (bottom).

A four-minute video was created to include five segments: four familiarization segments followed by one testing segment (see Figure 1). Each segment consisted of two scenes: an introduction scene and a demonstration scene. All five segments followed the same structure, except that the demonstration phase of the testing segment was differently structured as outlined below. Eight versions of the stimulus video were created for counterbalancing purposes (described below) but were otherwise identical to each other in length, behaviors, and speech.

#### Introduction

Each segment started with a scene depicting a female adult actor at the center of the screen. At the beginning of each segment, the actor introduced a problem (e.g., figuring out how to get a ball into the basket) without the presence of any physical tools for solving the problem.

#### Demonstration

In each segment, the introduction scene was followed by a scene of two live-action female adult informants solving the physical problem described in the introduction scene (e.g., constructing a simple machine like a ramp to get a ball into a basket; see Table 1 for a full list of problems and solutions and Supplementary Figure S1 for details). For the four familiarization segments, each informant took turns solving the problem consecutively (one character at a time rather than simultaneously), using either consistently high or low effort. The levels of effort were inversely related to the levels of efficiency of problem-solving solutions. During the familiarization segments, both informants were present; however, when one of the informants was speaking and solving the problem, the other informant remained still. In each familiarization segment, the *high effort/low efficiency informant* stated, “I am going to try really hard to solve this problem.” Then she solved the problem by exerting high effort and using an inefficient solution, such as creating and using a lever to get a ball into a basket. The *low effort/high efficiency informant* stated, “I do not have to try really hard to solve this problem.” Then she exerted comparably less effort to solve the same problem using an efficient solution, such as placing a ramp against the basket and rolling the ball into the basket. In each familiarization trial, the action sequence of the high effort/low efficiency character involved one additional step than that of the low effort/high efficiency character, resulting in an average difference of 13 s between the two characters across the four familiarization trials (*M_high_* = 24.6, *M_low_* = 11.2). The frequency and duration of social cues, such as neutral and smiling facial expressions, did not systematically differ between the characters. The problems and solutions were adapted from prior research examining children’s learning of problem-solving strategies from print and screen media (Brown and Kane, 1988; Richert and Smith, 2011; Schlesinger et al., 2016; Richert and Schlesinger, 2017). Across participants, we counterbalanced the pairing of actors to effort/efficiency type, the order in which the solutions were presented, and the screen position of each character (right vs. left side of the screen).

**Table 1:** Problems and solution action sequences during the familiarization phase.

Problem	High Effort/Low Efficiency Solution	Low Effort/High Efficiency Solution
Get a ball into a basket	(1) Place a block and (2) put a spoon on the block to create a lever and (3) use the lever to get the ball into the basket.	(1) Place a ramp against the basket and (2) roll the ball into the basket.
Get a ball from a distance	(1) Place a hook next to a long spoon, (2) tie the hook and the long spoon using a string, and (3) use the hook to retrieve the ball.	(1) Place a hook to a hole in a long spoon and (2) use the hook to retrieve the ball.
Make a short block as the same height as a tall block	(1) Pull out a balloon and an air pump, (2) inflate the balloon with the air pump, and (3) place the balloon underneath the small block.	(1) Pull out a new big block and (2) place the big block underneath the small block.
Stick two pieces of paper together	(1) Use a tape dispenser to pull and tear a piece of tape, (2) paste it between the two pieces, and (3) repeat the process two more times to bind the two pieces.	(1) Use a stapler to push a staple into the two pieces and (2) repeat the process two more times to bind the two pieces.

The testing segment was designed to measure children’s selective attention to each of the characters and children’s implicit learning preferences. During the testing segment, both informants solved the same problem (i.e., handing a bunch of marbles to a friend) simultaneously. Each informant used a different solution (towel, bowl) but showed the same amount of effort and efficiency without any statement being made. The type of tools (bowl, towel) was counterbalanced across participants. This task has been used in previous studies testing the transfer of problem-solving strategies in children within this age range (Richert et al., 2009). We counterbalanced the pairing of actors to effort/efficiency type, solution, and the side of presentation across participants.

#### Apparatus and setting

Children’s eye movements were captured using a Gazepoint (GP3) Desktop Eye Tracker. GP3 is a video-based remote eye-tracking system, which allows natural head movements of a user during the recording with an accuracy of 0.5–1° of visual angle based on manufacturer testing in ideal viewing conditions. The GP3 system records a range of gaze data from each eye (e.g., point of gaze) at a sampling rate of 60 HZ. The system allows for a 21° × 10° movement area with a range of ±13° depth of visual angle. Gazepoint Control software was used for calibration. Gazepoint Analysis software was used for data recording and analysis. A proprietary algorithm in the GazePoint Analysis software tool was used to identify fixations. Children’s eye movements were captured for the entire four-minute video. Eye gaze for the testing trial was evaluated separately for analyses.

The GP3 was set up in a room with controlled lighting and was affixed to a 54.6-cm ASUS LED monitor (screen resolution: 1,920 × 1,080). The video image was approximately 40° visual angle horizontally and 23° visual angle vertically. A chair was positioned approximately 65 cm from the eye-tracking camera and the display screen for optimal focus. An initial nine-point calibration procedure was followed by a four-point validation to determine the accuracy of the calibration in the lab. Spatial accuracy of eye-tracking calibrations averaged 1.4° (horizontal) by 0.5° (vertical) and did not differ by age.

### Procedure

The study was conducted by two researchers. The experimenter provided instructions and interacted with children in a laboratory testing room. The assistant controlled the equipment in an adjoining control room. The entire session lasted approximately 60 min.

#### Warm-up

Upon arrival, each family was escorted into a reception room. Parents completed consent forms and questionnaires while the experimenter played with children. The assistant explained the procedure to parents and answered questions before guiding children into the testing room. Parents sat in the reception room in which they could watch the session while completing a survey about demographic information such as caregiver’s education and income and child’s race and ethnicity as well as media use at home.

#### Cognitive development measures

Once children entered the testing room, the experimenter asked children to sit at a table. After completing the assent process, children’s general cognitive ability was assessed using the two standardized cognitive measurements from the Kaufman Assessment Battery for Children (KABC-II; Kaufman and Kaufman, 2004). In the Rebus subtest (general learning), children were taught the word or concept associated with each drawing and they then “read” aloud phrases and sentences consisting of these drawings. In the Conceptual Thinking subtest (simultaneous processing), children viewed a set of four pictures and identified the one that did not belong with the others. The total raw scores of the Rebus and Conceptual Thinking measures, ranged from 0 to 78 and 0 to 28, respectively, were converted to age-adjusted standardized scores, ranging from 0 to 19.

#### Visual attention during testing

After completing the cognitive measures, children moved to sit in front of the eye tracker. Children first watched a 2-min Sesame Street video while the experimenter set up the eye tracker. A nine-point calibration sequence followed the video. The calibration sequence was repeated until a reliable signal was obtained. Following successful calibration, a four-point validation sequence was presented to determine the accuracy of the calibration. Finally, children watched the stimulus video, during which their eye movements were recorded.

#### Problem-solving task

After viewing the stimulus video, children participated in a problem-solving task. Based on prior research (Brown and Kane, 1988; Richert and Smith, 2011; Schlesinger et al., 2016; Richert and Schlesinger, 2017), children’s selective learning of problem-solving strategies was measured by children’s choice of the solution presented by either informant in the testing segment of the stimulus video (see Figure 1). Children were given a pile of marbles and three possible solutions to gather all of the marbles: an upside-down bowl, a towel, and a large cooking spoon. Children were asked to find a way to hand all the marbles to the researcher at once. Children could use the solution presented by one of the informants by either (a) turning the bowl over, putting all of the marbles into the bowl, and handing the bowl to the researcher, or (b) putting all of the marbles into the towel, folding up the towel, and handing the folded towel to the researcher. Children could also use neither informant’s solution by choosing the large cooking spoon. Once children produced their first solution, they were prompted to “think of another way to get all the marbles.” After a second attempt, children were asked to “think back to the video” and to think about “whether anything from that video gives them another idea.” These prompts were adapted from prior research on children’s problem solving (Schlesinger et al., 2016). Children’s first solution attempt was coded as their problem-solving choice except for those who chose the distractor object (e.g., cooking spoon) or did not use any solution object (e.g., only used their hands) during their first attempt. In those cases, children’s solution during the following prompts was coded as their solution choice. Presentation of the stimulus video was counterbalanced across participants, such that 54% of children watched a testing video segment in which the high effort/low efficiency informant used the bowl and the low effort/high efficiency informant used the towel, and the remaining 46% of children watched a testing video segment in which the high effort/low efficiency informant used the towel and the low effort/high efficiency informant used the bowl.

#### Credibility ratings

Finally, children’s explicit judgments of character credibility were assessed. These credibility rating questions were adapted from prior studies on preschoolers’ selective social learning (Corriveau and Harris, 2009; Schlesinger et al., 2016). The experimenter presented the image of each informant and a circle scale that had five circles of different sizes. The circles were coded from 0 (smallest) to 4 (biggest), with a higher score indicating a higher credibility rating. First, children received two sets of questions as training for the circle scale. The first set of training questions focused on children’s positive answers (i.e., “What is your favorite color? How much do you like that color? Show me on the circles.”). The second set of training questions was about children’s negative answers (i.e., “Let us think about things you do not like. What food do not you like? How much do you like that food? Show me on the circles”). If children were unsure, the experimenter explained the scale again and repeated the questions to ensure that children were able to use the scale. Following the scale training, children were asked how good each informant was at solving problems using the five circles, resulting in character credibility scores ranging from 0 to 4.

### Data reduction

**Figure 2:**
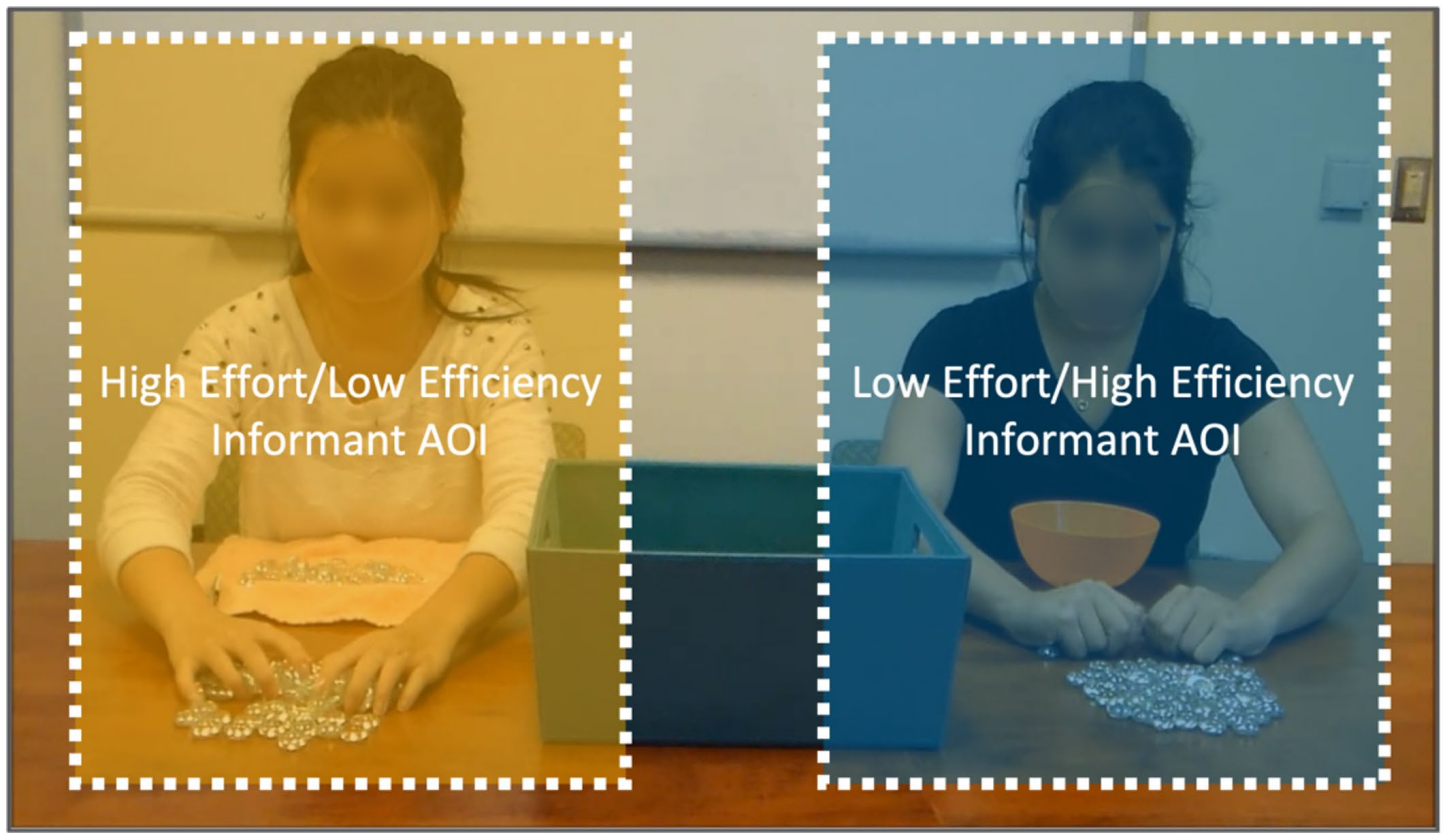
Example areas of interest (AOIs) during the testing trial.

Using Gazepoint Analysis software, the areas of interest (AOIs) for both informants were defined as two equal-sized rectangles. The AOI for the background was defined as the area that was not occluded by the AOIs for the two informants. Each rectangle includes one of the two informants on their respective sides of the screen (see Figure 2). Each AOI was approximately 12° visual angle horizontally and 20° visual angle vertically. We analyzed the total duration of visual fixations to the two AOIs (i.e., high effort/low efficiency informant and low effort/high efficiency informant).

Among the 70 children included in behavioral analyses, nine children (13%) did not provide any eye-tracking data due to child movements (e.g., as children leaned toward the screen or tilted their heads), equipment failures, and experimental errors (e.g., forgot to record), and therefore were not included in the analyses of the eye-tracking data. These nine children (*M* = 4.45, *SD* = 0.54; six boys and three girls) were significantly younger than children with eye-tracking data (*M* = 5.20, *SD* = 0.81; 30 boys and 31 girls), *t*(68) = − 2.68, *p* = 0.009, Cohen’s *d* = −0.96, 95% confidence interval (CI) = [−1.69, −0.23]. The two groups did not systematically differ by gender, Fisher’s exact test, *p* = 0.479, 95% CI = [0.39, 13.80], standardized cognitive development scores measured with the Rebus subtest, *t*(67) = −1.74, *p = 0*.087, *d* = −0.62, 95% CI = [−1.34, 0.10] as well as the Conceptual Thinking subtest, *t*(68) = −1.65, *p = 0*.103, *d* = −0.59, 95% CI = [−1.31, 0.13], and problem-solving solution choice, Fisher’s exact test, *p* = 0.494, 95% CI = [0.35, 12.12].

Additionally, based on *a priori* criteria, participants with less than 50% of eye-tracking data during the testing trial (due to inattention or moving the head out of the trackable range of the system) were excluded from data analyses involving eye movements (Thibaut and French, 2016). Thus, among the 61 children who provided eye-movement data, the mean proportion of gaze samples with usable gaze data was 79% (48 out of 61). These 48 children (*M_age_* = 5.15, *SD_age_* = 0.78; 24 boys and 24 girls) did not systematically differ from those with less than 50% eye-tracking data (*M_age_* = 5.38, *SD_age_* = 0.94; six boys and seven girls) on age, *t*(59) = −0.89, *p =* 0.376, *d* = −0.28, 95% CI = [−0.91, 0.35], gender, $\chi^{2}\left(1\right)$ = 0.01, *p* = 0.999, Cramer’s V < 0.001, standardized cognitive development scores measured using the Rebus subtest, *t*(58) = 0.78, *p* = 0.440, *d* = 0.24, 95% CI = [−0.39, 0.87] as well as the Conceptual Thinking subtest, *t*(59) = −1.55, *p* = 0.125, *d* = −0.49, 95% CI = [−1.12, 0.15], and solution choice, $\chi^{2}\left(1\right)$ = 0.18, *p* = 0.670, V = 0.05.

The variable of interest was the proportion of time children spent looking at the informants during the testing trial, where children watched the two informants demonstrate their unique solutions simultaneously. The proportion of gaze to the high effort/low efficiency informant was defined as children’s looking time to the high effort/low efficiency informant divided by children’s looking time to the entire screen, including both targets (both informants) and background. The proportion of gaze to the low effort/high efficiency informant was defined as children’s looking time to the low effort/high efficiency informant divided by children’s looking time to the entire screen.

### Analytical approach

Preliminary analyses included descriptive statistics and bivariate and partial correlations to determine covariates to be included in subsequent analyses. To test our three main research questions, we conducted the following analyses. First, we used a logistic regression model to predict preschoolers’ likelihood of choosing the low effort/high efficiency information (0 = using high effort/low efficiency informant’s solution; 1 = using low effort/high efficiency informant’s solution) as a function of age. Next, we conducted a linear mixed-effects model (effort given inversely related efficiency nested within participants) to test whether children’s proportion of looking to informants varies as a function of character effort with inversely related efficiency (0 = high effort/low efficiency, 1 = low effort/high efficiency), child age, and their interaction. Lastly, we used a linear mixed-effects model (effort given inversely related efficiency nested within participants) to predict children’s ratings of character credibility. This model included informant effort given inversely related efficiency (0 = high effort/low efficiency, 1 = low effort/high efficiency) as a within-subject predictor, age as a between-subject predictor, and their interaction. In all models described above, the age variable was treated as a continuous predictor and centered at the youngest age per model, and any necessary covariates were controlled. These models were estimated using the *glm* function from the stats package and the *lmer* function from the lme4 package (Bates et al., 2015) in the R software environment (Version 4.1.3; R Core Team, 2022).

## Results

### Preliminary analysis

Preliminary analyses indicated that children’s problem solving, gaze behavior, and credibility rating did not relate to child gender, race/ethnicity, cognitive skills, media use at home, or parent income and education. Thus, these variables were not considered further. However, children were more likely to use the bowl than the towel to solve the problem regardless of age (52 out of 70, 74%), *p* < 0.001, binomial test. Although tool type was counterbalanced across participants in this study, we addressed potential tool type effects by including tool type as a covariate in the model that included children’s problem-solving performance.

### Problem-solving solution choice as a function of age

Among the 70 children who completed the problem-solving task, more than half of the children (54%) selected the high effort/low efficiency informant’s solution, and the rest (46%) chose the low effort/high efficiency informant’s solution. A binomial test indicated that this difference was not significantly different from chance of 50%, *p* = 0.550.

A logistic regression model predicted children’s problem-solving solution choice (0 = using high effort/low efficiency informant’s solution; 1 = using low effort/high efficiency informant’s solution) based on age (centered at the youngest age, 3.67 years), controlling for tool type. Informant’s tool type was a significant predictor of problem-solving solution choice such that children were more likely to imitate the low effort/high efficiency informant when the informant used the bowl than the towel, *b* = 2.14, *SE* = 0.58, Wald *z* = 3.72, *p* < 0.001, odds ratio (OR) = 8.52, 95% CI = [2.89, 28.24].

**Figure 3:**
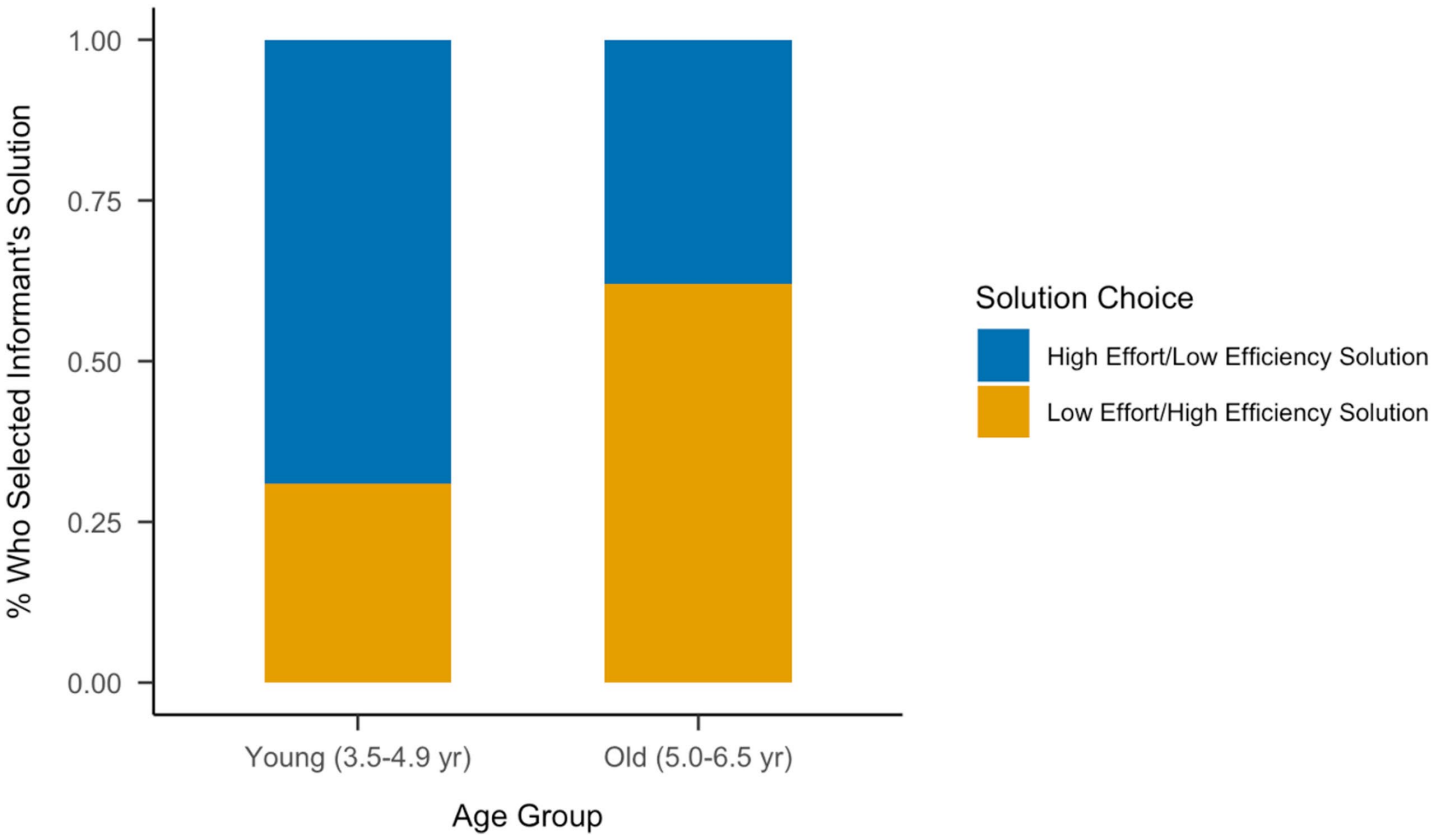
Proportion of children in each age group who selected either the high effort/low efficiency informant solution or the low effort/high efficiency informant solution during the problem-solving task.

After controlling for tool type, age was a significant predictor of the problem-solving solution choice, *b* = 0.76, *SE* = 0.37, *z* = 2.08, *p* = 0.037, OR = 2.15, 95% CI = [1.08, 4.62]. Thus, with increasing age, preschoolers were more likely to choose the low effort/high efficiency informant’s solution than the high effort/low efficiency informant’s solution. Similarly, when using a median split (median = 4.98 years) to define younger (3.5–5 years) and older (5–6.5 years) age groups, there was a significant difference between the age groups, $\chi^{2}\left(1\right)$ = 5.66, *p* = 0.017, Cramer’s V = 0.284. Specifically, 3.5–4.9-year-old children were more likely to choose the high effort/low efficiency informant’s solution (69%) than the low effort/high efficiency information’s solution (31%), and 5–6.5-year-old children were more likely to choose the low effort/high efficiency informant’s solution (62%) than the high effort/low efficiency information’s solution (38%; see Figure 3).

### Visual attention as a function of age

Among the 48 children who provided eye gaze data, the overall mean proportion of looking time was slightly greater to the high effort/low efficiency informant (*M* = 0.42, *SD* = 0.18) than to the low effort/high efficiency informant (*M* = 0.39, *SD* = 0.19), but this difference was not statistically significant, *t*(47) = 0.64, *p* = 0.527, Cohen’s *d* = 0.17, CI = [−0.37, 0.71].

Table 2 presents fixed effects for the full model predicting children’s proportion of gaze to informants as a function of effort with inversely related efficiency, age, and their interaction. In Table 2, the effect of effort with inversely related efficiency represents the difference between the proportion of looking to the low effort/high efficiency informant and the proportion of looking to the high effort/low efficiency informant at the youngest age of 3.85 years. There was a significant effect of informant effort with inversely related efficiency for the youngest children such that these children spent less time looking to the low effort/high efficiency informant than the high effort/low efficiency informant, *b* = −0.16, *p* = 0.027.

**Table 2:** Fixed effects from the linear mixed-effects model predicting the proportion of gaze to the target AOIs as a function of informant effort/efficiency and child age.

Predictors	*b* (SE)	*t*	*df*	*p*	95% CI
(Intercept)	0.47 (0.05)	9.01	92	<0.001^***^	[0.36, 0.57]
Effort (Low)/Efficiency (High)	−0.16 (0.07)	−2.25	92	0.027^*^	[−0.31, −0.02]
Age	−0.03 (0.03)	−1.04	92	0.334	[−0.10, 0.03]
Effort (Low)/Efficiency (High) × Age	0.10 (0.05)	2.1	92	0.038^*^	[0.01, 0.20]

Informant effort/efficiency was dummy-coded: high effort/low efficiency informant (reference category) and low effort/high efficiency informant. Age was a continuous variable, centered at the youngest age of 3.85 years. ^*^p < 0.05, ^***^p < 0.001.

**Figure 4:**
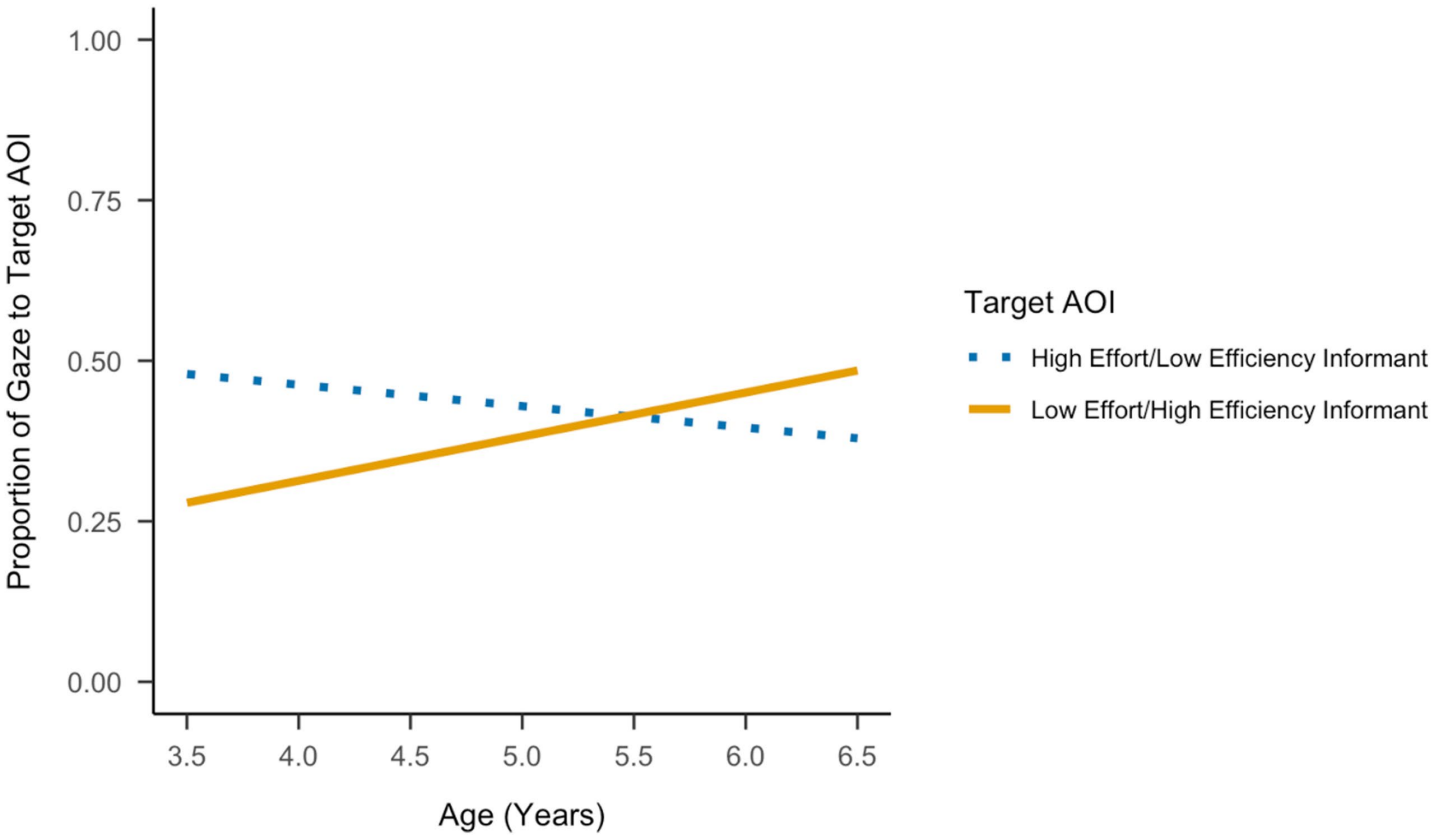
Predicted proportion of gaze to the target AOIs during testing as a function of informant (high effort/low efficiency, low effort/high efficiency) and child age.

The remaining set of effects in Table 2 shows how children’s proportion of looking to the high effort/low efficiency informant changed with age and whether the age effect was moderated by informant effort with inversely related efficiency. There was a negative but not significant age effect on children’s attention to the high effort/low efficiency informant, *b* = −0.03, *p* = 0.334. However, there was a significant interaction between informant effort with inversely related efficiency and age, *b* = 0.10, *p* = 0.038. That is, the age effect was modified by informant effort with inversely related efficiency such that the proportion of time spent looking at the low effort/high efficiency informant gradually increased with age between 3.5 and 6.5 years (see Figure 4).

To identify whether age had an effect on the proportion of time spent looking at the low effort/high efficiency informant, we conducted an analogous *post hoc* model, recentering effort/efficiency to change the reference group from the high effort/low efficiency informant to the low effort/high efficiency informant. This *post hoc* model showed a positive age effect on children’s attention to the low effort/high efficiency informant, *b* = 0.07, *t*(92) = 2.00, *p* = 0.048. Next, we recentered age at its maximum instead of its minimum to examine the effect of effort with inversely related efficiency among those at the oldest end of the age range (6.62 years). The oldest children spent slightly more time looking at the low effort/high efficiency informant than the high effort/low efficiency informant, but this difference was not statistically significant, *b* = 0.12, *t*(92) = 1.46, *p* = 0.147.

### Credibility judgments as a function of age

Among the 69 children who provided credibility ratings, the overall mean credibility attributed to the high effort/low efficiency informant (*M* = 3.19, *SD* = 1.35) was higher, but only marginally, than the overall mean credibility of the low effort/high efficiency informant (*M* = 2.76, *SD* = 1.42), *t*(68) = 1.83, *p* = 0.072, Cohen’s *d* = 0.31, CI = [−0.03, 0.66]. Table 3 presents fixed effects for the full model predicting children’s credibility ratings as a function of effort with inversely related efficiency, age, and their interaction. In this model, none of the predictors were significant, *p* ≥0.245.

**Table 3:** Fixed effects from the linear mixed-effects model predicting credibility ratings as a function of informant effort/efficiency and child age.

Predictors	*b* (SE)	*t*	*df*	*p*	95% CI
(Intercept)	3.09 (0.34)	9.01	135	<0.001^***^	[2.41, 3.76]
Effort (Low)/Efficiency (High)	−0.56 (0.48)	−1.17	135	0.245	[−1.51, 0.39]
Age	0.07 (0.21)	0.34	135	0.732	[−0.34, 0.48]
Effort (Low)/Efficiency (High) × Age	0.09 (0.29)	0.31	135	0.755	[−0.48, 0.67]

Informant effort/efficiency was dummy-coded: high effort/low efficiency informant (reference category) and low effort/high efficiency informant. Age was a continuous variable, centered at the youngest age of 3.67 years. CI, confidence interval. ^***^p < 0.001.

## Discussion

The purpose of the current study was to examine if preschoolers selectively allocate attention to and learn from a character who tried hard to solve problems using inefficient solutions (*high effort/low efficiency*) and a character who could easily solve problems using efficient solutions (*low effort/high efficiency*). Consistent with prior studies (Nicholls, 1978; Nicholls and Miller, 1984; Folmer et al., 2008), there was an age-related increase in children’s likelihood of choosing the low effort/high efficiency character’s solution. Younger children (3.5–4.9 years) chose to learn from the character who worked hard and inefficiently compared to the character who did not have to exert the same high amount of effort and work efficiently. Older children (5.0–6.5 years), however, displayed an opposite pattern such that they were more likely to learn from the character who effortlessly and efficiently problem-solved. Preschoolers’ visual attention reflected a similar change as in their problem-solving solution choice. That is, younger children paid more attention to the high effort/low efficiency character than the low effort/high efficiency character, but there was an age-related increase in children’s attention to the low effort/high efficiency character. However, children’s credibility ratings did not significantly differ by either character effort with inversely related efficiency or child age. Together, these findings suggest that character effort that inversely related efficiency influences children’s encoding processes and partially their retrieval as children demonstrated implicit, but not explicit, learning preferences.

We found that younger children (3.5–4.9 years) were more likely to choose the high effort/low efficiency character’s solution than the low effort/high efficiency character’s solution. In line with prior research showing that even children under age 5 years attend to confidence as cues to infer who will offer relevant information (Jaswal and Malone, 2007), preschoolers in the current study recognized character effort that is inversely related to efficiency as a meaningful social cue and demonstrated an implicit learning preference for the character who worked hard. This finding is also consistent with the classic view on children’s reasoning about effort, which posits that younger children believe in a positive relation between effort and ability (Nicholls, 1978; Nicholls and Miller, 1984; Folmer et al., 2008).

However, this pattern changed with age, suggesting a shift in children’s perception of character effort in early childhood. Older preschoolers implicitly expressed their learning preferences toward the low effort/high efficiency character over the high effort/low efficiency character by endorsing the low effort/high efficiency character’s solution. This developmental shift was observed earlier in the current study than what was claimed in the classic work on children’s reasoning about effort and ability (e.g., Nicholls, 1978). Based on the findings from the current study, older preschoolers appear to reason about the compensatory nature of the relation between effort and ability, which is similar to the responses of the older children and adults in prior research (Nicholls, 1978; Nicholls and Miller, 1984). Therefore, these results partially support the claim that preschoolers can make adult-like inferences about effort and ability (Wimmer et al., 1982; Heyman et al., 2003; Heyman and Compton, 2006).

Another key finding from this study is that younger children looked longer at the high effort/low efficiency informant, but children’s looking to the low effort/high efficiency informant increased with age. This pattern is compatible with children’s performance in the problem-solving task. This consistency between children’s eye movements and behaviors aligns with previous research indicating selectivity in 3.5–4-year-old children’s eye movements as well as in their word learning (Sobel et al., 2012). Given that children’s cognitive control skills such as working memory and inhibitory control rapidly increase between 3 and 5 years of age (Carlson, 2005; Garon et al., 2008), developmental improvements in these cognitive skills appear to help preschoolers by reliably guiding attention using top-down processes driven by children’s perceptions of informants’ effort that is presented to be inversely related to efficiency. As individual differences in general learning and simultaneous processing skills were not related to children’s gaze patterns, it appears that children’s selective attention is not entirely dependent on children’s cognitive skills, at least as they were measured in the current study. It is possible that children (3.5–6.5 years) included in this study had a sufficient level of cognitive functioning to demonstrate selectivity in the given social learning task, thus resulting in the selective visual attentional patterns independent from children’s cognitive skills. Together, these results suggest that character effort may influence the dynamics of selective social learning in real time through the ways in which children encode information from characters.

However, there was no significant age-related change in children’s explicit judgments of character credibility. The differences between implicit and explicit measures of selective social learning have been reported in some prior studies (Bascandziev and Harris, 2016; Corriveau et al., 2016; Leech et al., 2019). One possibility is that preschoolers continue to perceive both characters as credible as long as their effort results in successful outcomes. In prior research, observing an adult model’s hard work increased children’s persistence when a positive, but not negative, outcome was expected (Leonard et al., 2020). The credibility cues in the form of varying levels of character effort combined with reversely related efficiency levels might have been too subtle to influence children’s explicit judgments of credibility, which may require stronger cues (e.g., failure) to be associated with effort. This asymmetry between the implicit and explicit measures is a worthy of further investigation as it may help explain the differences among the prior findings on preschoolers’ understanding of high and low effort characters.

Although children’s credibility ratings did not differ by character effort with reversely related efficiency or child age, children’s implicit learning preferences may have moderated these associations. To further explore potential associations between the implicit and explicit measures, we conducted a follow-up exploratory analysis on correlations among assessments (see Supplementary Table S1). The results showed that whether children selected the high or low effort character’s solution was associated with children’s credibility judgment of the low effort/high efficiency character, but not the high effort/low efficiency character. These patterns suggest possible interactions among character effort with reversely related efficiency, child age, and child implicit learning preferences. The current study was not designed nor sufficiently powered to examine these interactions, but future research should include larger samples to test how multiple factors interact and influence children’s explicit judgments about the credibility of both high and low effort characters with different levels of efficiency.

Overall, these findings indicate that preschoolers view effort that is reversely related to efficiency as a meaningful social cue. Even younger children (3.5–4.9-year-old children) differentiated the high effort/low efficiency character from and the low effort/high efficiency character, demonstrating preferences by higher attention to and greater learning of problem solving from the high effort/low efficiency character than the low effort/high efficiency character. Critically, there was a shift in children’s selective learning from the high and low effort characters with contrasting efficiency levels during early childhood. Older children showed preferences toward the low effort/high efficiency informant’s solution when problem solving, which reflects an adult-like reasoning pattern. Notably, we found age-related changes in the context where the two characters were paired. Prior research on effort allocation suggests preschoolers’ rational yet optimistic thoughts about effort regardless of age (Leonard et al., 2020). Our results may differ from what has been observed in prior research because we asked children to compare the high and low effort characters with reversely related efficiency levels rather than presenting each character in isolation. Perhaps preschoolers’ implicit learning preferences become more apparent when having a low effort/high efficiency character presented as a clear alternative to a high effort/low efficiency character rather than each character alone (Vanderbilt et al., 2014). Researchers have found social comparison behaviors among children even in preschools, a relatively non-competitive pedagogical environment compared to elementary or secondary schools (Mosatche and Bragonier, 1981; Chafel, 1987). Thus, preschoolers may be sensitive to the social contexts in which varying levels of effort are present by parents, siblings, peers, or media characters. Importantly, preschoolers in this study did not distinguish the high and low effort characters with reversely related efficiency in their explicit judgments about character credibility, suggesting that early childhood may be a unique and malleable period in the formation of credibility beliefs about effort and efficiency. Together, the current findings suggest that the later preschool years (between 5 and 6.5 years) may be a particularly critical time in which to deliver messages about the value of hard work, especially in social contexts where comparisons are often present if the goal is to promote a growth mindset in early childhood (Dweck, 2006).

As the stimuli were presented on video, these findings also have implications for children’s social learning from screen media (Richert et al., 2011). Newer educational media for children is going beyond teaching literacy and numeracy by developing curricula to teach skill sets (e.g., read-to-learn skills, confidence, and attention) to prepare children to jump into school ready and excited to learn (Hirsh-Pasek et al., 2015; Choi, 2021). This curriculum is primarily being funneled through media characters with the expectation that young children will recognize and assimilate traits of a media character into themselves. Prior research has indicated that young children’s beliefs about media characters’ credibility and reality status are related to their learning from those characters (Schlesinger et al., 2016; Richert and Schlesinger, 2017). The current study reveals that young children also recognize effort when demonstrated by on-screen models and use character’s effort with reversely related efficiency as a meaningful social cue to guide their attention and learning.

### Limitations and future directions

A primary limitation of the current study is the percentage of children who did not present useable eye-tracking data. Notably, the children who did not provide any eye movement data were significantly younger than the participants included in data analysis. Although it is common in eye-tracking studies not to have useable data on all child (or even adult) participants for a variety of reasons (e.g., reflections on eyeglasses and body movements; Duchowski, 2007), the current findings specifically on age differences in children’s visual attention patterns should be interpreted as generalizable primarily for children who could and would maintain visual attention to stimuli for long enough to provide useable data (in this case, 50% looking time during the selective attention trial).

A secondary limitation of the current study is that there was an unexpected effect of the type of tools used by the characters. We confirmed that the general pattern of the findings did not change regardless of the inclusion of tool type in the models, but we decided to include this in the model to statistically control any potential influence of this variable. Future studies should take into account this shortcoming.

A third limitation of the current study is that we covary effort and efficiency by manipulating both the verbal statement and the type and duration of action for the characters. In this study, the characters showed efficiency levels that were inversely related to effort levels, using different strategies with varying durations to allow for a distinct comparison between the two characters. This confound was intentionally introduced in this study to help young children avoid sources of confusion identified in prior studies (e.g., children’s difficulty in understanding a combination of low effort and high outcome without levels of ability as an explanation; Wimmer et al., 1982). Thus, the current study does not answer whether effort, efficiency, or the two in combination would be most salient for children’s perception of characters. As effort and efficiency are not always mutually exclusive, further studies should disentangle these components in order to fully examine the interplay between effort and efficiency.

In addition, in the current study, both characters’ problem-solving attempts resulted in success in order to distinguish effort from other social cues such as confidence or accuracy. Given that preschoolers learn to persist after observing others’ hard work that leads to success but not when it results in failure (Leonard et al., 2020), it would be an important next step to examine how the match and mismatch between effort, efficiency, and outcome would affect children’s selective social learning. When it comes to educational media for young children, children regularly encounter child-like main characters—who are curious but not always skillful—gain knowledge and skills through interaction with more advanced others. How would children learn from the inquisitive yet often unskilled characters that present varying levels of effort? It remains to be seen the extent to which character effort and efficiency influences children’s learning from ecologically valid media characters who display complex and dynamic social cues.

Lastly, children’s own level of effort and efficiency was not measured in the present study. One possibility is that preschoolers’ preference to watch or use the solution provided by either the high or low effort character would lead children themselves to work hard or effortlessly in different tasks. Prior research found that infants as young as 15 months learned to persist from an adult experimenter who repeatedly tried to achieve a goal, but not from an adult experimenter who accomplished that goal quickly (Leonard et al., 2017). Further, storybooks designed to emphasize the value of effort were shown to help children persist on a new task (Haber et al., 2022). It would be an important future direction to examine the extent to which preschoolers’ understanding of character effort is related to children’s learning and generalization of perseverance behaviors from the characters.

## Conclusion

Together, our findings suggest that character effort with reversely related efficiency serves as a meaningful social cue to guide preschoolers’ attentional and implicit learning preferences, the specific patterns of which are modified by age. These findings add to our understanding of children’s social learning by informing when and how children learn from high effort/low efficiency and low effort/high efficiency on-screen characters in early childhood. It additionally begins to fill a gap in the existing literature by investigating visual attention to understand the ongoing dynamics of selective social learning. Our findings corroborate the importance of considering developmental changes and the social nature of children’s learning in designing screen media for early childhood education. Studying how children perceive effort in their early years can offer insights into the unique characteristics and needs of individuals at specific developmental stages. In light of the life-long significance of one’s belief about effort, this line of work will serve as a critical foundation for investigating longitudinal changes and developing tailored interventions.

## Data availability statement

The raw data supporting the conclusions of this article will be made available by the authors, without undue reservation.

## Ethics statement

The studies involving human participants were reviewed and approved by the University of California, Riverside. Written informed consent to participate in this study was provided by the participants’ legal guardian/next of kin.

## Author contributions

RR conceptualized the study in collaboration with JF. RR secured funding. RR, JF, and MS designed the study. MS made the stimulus video. KC and MS collected data. KC analyzed the data and wrote the first draft of the manuscript. MS, RR, and JF provided substantial feedback on data analysis and writing. All authors contributed to the article and approved the submitted version.

## Funding

This research presented in the manuscript was funded by Grant No. DRL-1252146 from the National Science Foundation to RR. Any opinions, findings, and conclusions or recommendations expressed in this material are those of the author(s) and do not necessarily reflect the views of the National Science Foundation. This article’s publication was funded by the Virginia Tech Open Access Subvention Fund and by the Niles Research Grant from the Virginia Tech College of Liberal Arts and Human Sciences to KC.

## Conflict of interest

The authors declare that the research was conducted in the absence of any commercial or financial relationships that could be construed as a potential conflict of interest.

## Publisher’s note

All claims expressed in this article are solely those of the authors and do not necessarily represent those of their affiliated organizations, or those of the publisher, the editors and the reviewers. Any product that may be evaluated in this article, or claim that may be made by its manufacturer, is not guaranteed or endorsed by the publisher.
