# Supplementary material for: Preschoolers’ attention to and learning from on-screen characters that vary by effort and efficiency: An eye-tracking study
Source: Front Psychol. 2022 Dec 15;13:1011172. doi: 10.3389/fpsyg.2022.1011172 (PMC9798126; doi:10.3389/fpsyg.2022.1011172)
Supplement: Supplementary file 1 [file Table_1.PDF]

Table S1. Zero-Order Bivariate Correlations Between Assessments (Below Diagonal) and Partial Correlations Controlling for Child Age (Above Diagonal)

	1.	2.	3.	4.	5.	6.
1. Child age (months)	–	–	–	–	–	–
2. Problem-solving solution choice (low effort/high efficiency)	.28*	–	-.04	.05	-.04	.29*
3. Proportion of gaze to the high effort/low efficiency character	-.12	-.07	–	-.76***	.22	.05
4. Proportion of gaze to the low effort/high efficiency character	.25	.12	-.76**	–	-.11	-.01
5. Credibility rating of the high effort/low efficiency character	.04	-.02	.21	-.09	–	-.01
6. Credibility rating of low effort/high efficiency character	.09	.30*	.05	-.01	-.01	–

Note. Variables included child age (months), problem-solving solution choice (0 = high effort/low efficiency character's solution, 1 = low effort/high efficiency character's solution), proportion of gaze to the high effort/low efficiency character, proportion of gaze to the low effort/high efficiency character, credibility rating of the high effort/low efficiency character, and credibility rating of the low effort/high efficiency character. The correlation matrix included Pearson correlations for continuous variable pairs (e.g., child age ~ proportion of gaze to the high effort/low efficiency character) and point-biserial correlations for continuous-dichotomous pairs (e.g., child age ~ problem-solving solution choice).

$^{*}p < .05$, $^{**}p < .01$, $^{***}p < .001$.
