# Supplementary material for: Preschoolers’ attention to and learning from on-screen characters that vary by effort and efficiency: An eye-tracking study
Source: Front Psychol. 2022 Dec 15;13:1011172. doi: 10.3389/fpsyg.2022.1011172 (PMC9798126; doi:10.3389/fpsyg.2022.1011172)
Supplement: Supplementary file 2 [file Image_1.PDF]

Figure S1. Screenshots and Descriptions of Problems and Solution Action Sequences During the Familiarization Phase

**Problem**	**High Effort/Low Efficiency Solution**	**Low Effort/High Efficiency Solution**
Get a ball into a basket	(1) Place a block and (2) put a spoon on the block to create a lever and (3) use the lever to get the ball into the basket. 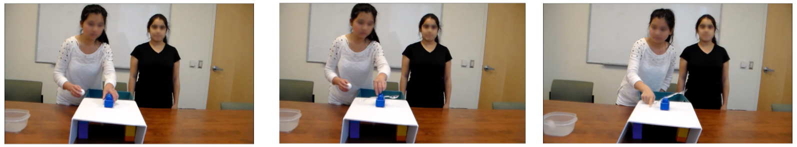	(1) Place a ramp against the basket and (2) roll the ball into the basket. 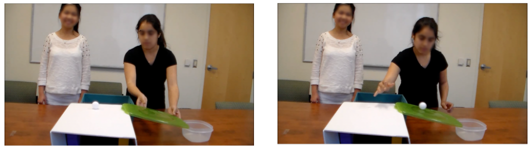
Get a ball from a distance	(1) Place a hook next to a long spoon, (2) tie the hook and the long spoon using a string, and (3) use the hook to retrieve the ball. 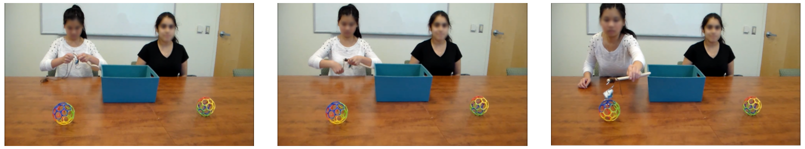	(1) Place a hook to a hole in a long spoon and (2) use the hook to retrieve the ball. 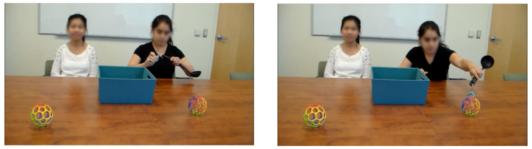
Make a short block as the same height as a tall block	(1) Pull out a balloon and an air pump, (2) inflate the balloon with the air pump, and (3) place the balloon underneath the small block. 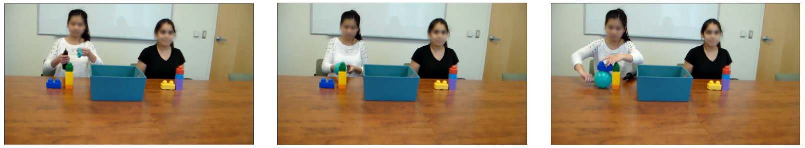	(1) Pull out a new big block and (2) place the big block underneath the small block. 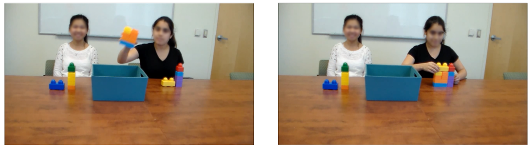
Stick two pieces of paper together	(1) Use a tape dispenser to pull and tear a piece of tape, (2) paste it between the two pieces, and (3) repeat the process two more times to bind the two pieces. 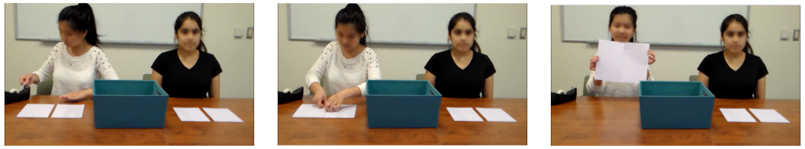	(1) Use a stapler to push a staple into the two pieces and (2) repeat the process two more times to bind the two pieces. 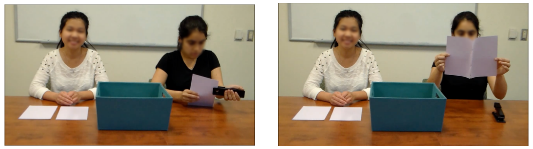
